# Transcriptome Investigation and In Vitro Verification of Curcumin-Induced HO-1 as a Feature of Ferroptosis in Breast Cancer Cells

**DOI:** 10.1155/2020/3469840

**Published:** 2020-11-19

**Authors:** Ruihua Li, Jing Zhang, Yongfeng Zhou, Qi Gao, Rui Wang, Yurong Fu, Lianwen Zheng, Hao Yu

**Affiliations:** ^1^College of Animal Science, Jilin University, Changchun 130062, China; ^2^Key Laboratory of Zoonosis Research, Ministry of Education, Jilin University, Changchun 130062, China; ^3^Biological Emergency and Clinical POCT Key Laboratory, Beijing 102600, China; ^4^Reproductive Medical Center, Department of Obstetrics and Gynecology, The Second Hospital of Jilin University, Changchun 130022, China

## Abstract

Ferroptosis is a form of oxidative cell death and has become a chemotherapeutic target for cancer treatment. Curcumin (CUR), a well-known cancer inhibitor, significantly inhibits the viability of breast cancer cells. Through transcriptomic analysis and flow cytometry experiments, it was found that after 48 hours of treatment of breast cancer cells at its half maximal inhibitory concentration (IC50), curcumin suppressed the viability of cancer cells via induction of ferroptotic death. Use of the ferroptosis inhibitor ferrostatin-1 and the iron chelator deferoxamine rescued cell death induced by curcumin. Furthermore, in subsequent cell validation experiments, the results showed that curcumin caused marked accumulation of intracellular iron, reactive oxygen species, lipid peroxides, and malondialdehyde, while glutathione levels were significantly downregulated. These changes are all manifestations of ferroptosis. Curcumin upregulates a variety of ferroptosis target genes related to redox regulation, especially heme oxygenase-1 (HO-1). Using the specific inhibitor zinc protoporphyrin 9 (ZnPP) to confirm the above experimental results showed that compared to the curcumin treatment group, treatment with ZnPP not only significantly improved cell viability but also reduced the accumulation of intracellular iron ions and other ferroptosis-related phenomena. Therefore, these data demonstrate that curcumin triggers the molecular and cytological characteristics of ferroptosis in breast cancer cells, and HO-1 promotes curcumin-induced ferroptosis.

## 1. Introduction

Breast cancer is the most common invasive cancer in women and the second most common cause of death [1]. Globally, approximately 2.1 million new breast cancer cases were diagnosed in 2018, accounting for one-quarter of cancer cases in women [2]. According to the North American Association of Central Cancer Registries (NAACCR) criteria, the breast cancer subtypes are defined as HR+/HER2-, HR+/HER2+, HR-/HER2+, and HR-/HER2- [3]. Due to the poor prognosis and tumor heterogeneity of breast cancer, no clear molecular target has been identified, making the recovery of breast cancer patients very challenging [4]. In addition, fewer than 30% of women with metastatic triple negative breast cancer (TNBC) survive 5 years [5]. Therefore, in addition to the known effective molecular targets of classical chemotherapy treatment, the search for new targets among natural drugs with extensive anticancer effects is expected to become a feasible strategy for the safe treatment of breast cancer [6].

Curcumin has been widely and safely consumed for hundreds of years as a natural food color, and preclinical studies have shown its potential applications in both pharmacology and cancer treatment [7]. Curcumin was first discovered by Vogel and Pelletier in turmeric rhizomes (turmeric) and is chemically referred to as diferuloylmethane [8]. Previous studies have shown that curcumin has antiproliferative and proapoptotic effects in pancreatic cancer cells [9], prostate cancer cells [10], and malignant mesothelioma cell lines [11]. Curcumin not only effectively removes active oxygen but also activates antioxidant response elements to inhibit active oxygen-induced lipid peroxidation [12]. Interestingly, it has been shown that curcumin inhibits the production of reactive oxygen species at low concentrations but induces the production of reactive oxygen species at high concentrations [13]. Depending on the cell type, curcumin may exhibit both antioxidant and prooxidant effects [14]. In addition, numerous studies have shown that curcumin upregulates the expression of HO-1 in a variety of cells. Shi and Li showed that HO-1 expression was upregulated in a dose- and time-dependent manner after treatment of neuroblastoma with curcumin [15]. Recent studies have shown that upregulation of HO-1 promotes the degradation of heme and the synthesis of ferritin, altering the iron distribution in cells. Enhanced HO-1 expression can increase or induce ferroptosis by promoting iron accumulation and reactive oxygen species (ROS) production [16], which means that curcumin is closely related to ferroptosis through its effects on HO-1.

Inducing direct cytotoxicity in cancer cells is one of the main goals of anticancer treatments. In general, apoptosis is considered the major form of cytotoxicity and is through to be required for tumor regression and sustained clinical remission [17]. Ferroptosis is a unique iron-dependent form of nonapoptotic cell death characterized by the accumulation of intracellular iron, which leads to the overproduction of ROS, decreased glutathione (GSH) levels, and lipid peroxidation [18, 19]. Recently, regulating mast cell processes has been used in a chemotherapy-based strategy for cancer treatment, and several drugs have been shown to trigger cell ferroptosis by acting on system X_c_^−^, glutathione peroxidase 4 (GPX4), and ferritin degradation through autophagy [20, 21]. Interestingly, curcumin can regulate the intracellular redox response and, as explained above, also induces the high HO-1 expression in cells, which may cause changes in intracellular ferritin. Therefore, a discussion of whether the anticancer effects of curcumin are the basis for induction of ferroptosis is worthwhile.

Curcumin affects a variety of molecular targets and signaling pathways, and bioavailability-enhanced curcumin preparations are administered to patients with breast cancer, in whom they have been observed to inhibit systemic inflammation and significantly improve the quality of life in these patients [11]. Therefore, the purpose of this study was to accurately assess the possible gene pathways targeted by curcumin in breast cancer cells using RNA sequencing and bioinformatics analysis and to explore and validate the therapeutic potential of curcumin in representative major breast cancer cell lines.

## 2. Materials and Methods

### 2.1. Chemicals and Reagents

Antimycotics (#15240062), fetal bovine serum (FBS, #10091148), RPMI 1640 medium (#A1049101), and Dulbecco's modified Eagle's high glucose medium (#11965118) were purchased from HyClone (HyClone, Logan, UT, USA). Cell Counting Kit-8 (CCK-8, #CK04) was obtained from Dojindo (Dojindo Laboratories, Kumamoto, Japan). The malondialdehyde (MDA) assay kit (#A003-1-2) and reduced glutathione (GSH) assay kit (#A006-2-1) were purchased from Jiancheng (Jiancheng, Nanjing, China). The DAPI staining solution kit (#MA0127) and zinc protoporphyrin-9 (#MB4231) were purchased from Meilui (Meilui, Dalian, China). Liperfluo (#L248) was purchased from Dojindo (Dojindo Laboratories, Shanghai, China). 2′,7′-Dichlorodihydrofluorescein diacetate (DCFH-DA, #S0033S), Annexin V (#C1062M-1), and Propidium Iodide (PI, #C1062M-3) were purchased from Beyotime (Beyotime, Shanghai, China). For western blot analysis, antibodies against HO-1 (#ab13243), GPX4 (#ab40993), Nrf2 (#ab137550), and *β*-actin (#ab8226) were purchased from Abcam (Abcam, Cambridge, MA). The Iron Assay Kit (#MAK025), dimethyl sulfoxide (DMSO, #D2650), curcumin (#C1386), z-VAD-fmk (#V116), ferrostatin-1 (#SML0583), and deferoxamine (#D9533) were purchased from Sigma (Sigma, St Louis, USA). These compounds were dissolved in DMSO (at final concentrations less than 0.1% (*v*/*v*)).

### 2.2. Cell Culture

The breast cancer cells used in this study were obtained from American type culture collection (ATCC). The human breast adenocarcinoma-derived MCF-7 cell line was cultured in Dulbecco's modified Eagle's high glucose medium supplemented with 10% FBS, 100 UI/mL penicillin, and 100 *μ*g/mL streptomycin. The human TNBC cell line MDA-MB-231 was cultured in RPMI 1640 medium supplemented with 10% FBS, 100 UI/mL penicillin, 100 *μ*g/mL streptomycin, and 2 mM glutamine. Cells were maintained in a humidified incubator at 37°C and 5% CO2. Cultures were tested periodically and confirmed to be mycoplasma-free.

### 2.3. Treatment Methods for Cells

Curcumin was dissolved in DMSO at a concentration of 50 mM for storage and diluted to specific concentrations in cell culture medium for cell treatments. ZnPP was dissolved in DMSO to a concentration of 6 mM and diluted to 10 *μ*M in cell culture medium for cell treatments. The final concentration of DMSO in the above-prepared treatment solutions was less than 0.1% (*v*/*v*). During treatment, the same volume of DMSO was added to the control groups. The curcumin stock solution was diluted in cell culture medium to concentrations of 5, 10, 20, 40, 60, 80, 100, 120, and140 *μ*M, and the same volume of DMSO was added to the control groups. When the confluence of breast cancer cells in the culture dish reached more than 80%, cell viability was tested after 48 hours of treatment with the above concentrations. The IC20 and IC50 values were calculated using GraphPad Prism7.0.

### 2.4. Treatment Method of Inhibitor

Culture the cells in a 6-well plate, and when the degree of polymerization of the cells in the culture dish reaches 80%, preincubate the breast cancer cells with 50 *μ*M z-VAD-fmk, 1 *μ*M ferrostatin-1, and 50 *μ*M deferoxamine, respectively, for 2 h. Then, the cells were treated with curcumin at IC50 concentration for 24 h or 48 h.

### 2.5. Cell Viability Assay

Cell viability was determined by the Cell Counting Kit-8 (CCK-8; Dojindo, Tokyo, Japan). Cells were seeded into 96-well plates at a density of 0.2 × 10^4^ cells per well, and after curcumin treatment of breast cancer cells for 24 h or 48 h, 10 *μ*L CCK-8 reagent was added to each well. Plates were incubated for 1 hour at 37°C, and the absorbance value (OD) of each well was measured at 450 nm according to the manufacturer's instructions.

### 2.6. RNA Sequencing

MCF-7 cells were treated with vehicle or 40 *μ*M curcumin for 48 h, and MCF-MB-231 cells were treated with vehicle or 50 *μ*M curcumin for 48 h. Breast cancer cells were collected and washed with cold PBS twice. According to the TriPure protocol, total RNA was extracted and resuspended in diethyl pyrocarbonate. After validating its integrity and purity, qualifying RNA was subjected to PCR amplification for the construction of a cDNA library. Raw data were filtered to remove low-quality sequences that might affect data quality and subsequent analysis. The cDNA library was sequenced using an Illumina HiSeq™ 300 platform. Raw sequence data were filtered using HISAT to remove low-quality sequences and adaptor reads. After quality trimming, Bowtie (1.1.2) was used to map pure reads to the *V. vinifera* reference genome using standard mapping parameters. Data that could be mapped to the reference genome were >100 bp in length and contained <2 mismatched reads. SAM tools and BamIndexStats were used to calculate the gene expression and reads per kilobase per million (RPKM). Gene expression annotation was performed using the Cufflinks software, and all the parameters were set to default values. The BioProject accession of this study is PRJNA613560.

### 2.7. K-Means Heat Map Clustering and Pathway Enrichment Analyses

K-Means heat map clustering was performed using the IDEP website (http://bioinformatics.sdstate.edu/idep/). KEGG pathway enrichment analyses were performed to determine upregulated and downregulated genes in each comparison group from the heat map cluster using the ClusterProfiler package [22].

### 2.8. Gene Set Enrichment Analysis (GSEA) and PPI Network Diagrams

GSEA analysis was based on NetworkAnalyst, an integrative approach for protein–protein interaction network analysis and visual exploration, and the GSEA results were used to correlate the gene signature with the effects of curcumin. The normalized enrichment score (NES) is the primary statistic for examining gene set enrichment analysis results, and the nominal *P* value estimates the significance of the enrichment score. A gene set with a nominal *P* ≤ 0.05 was considered to be significantly enriched in the genes identified. PPI network diagrams were generated using the Cytoscape 3.7.2 software for visualization and analysis of biological networks.

### 2.9. Staining with Annexin V and PI

Ferroptosis was assessed following the method described by Chen et al. [23]. After treatments, cells were collected and stained with Annexin V-FITC reagent and PI followed by analysis by flow cytometry. The percentage of dead cells was quantified using the FlowJo 10.5 software.

### 2.10. Fluorescence Measurements of Intracellular Oxidants

DCFH-DA was used to detect ROS production in cells. The fluorescence of each labelled penetrant significantly increased after oxidation in cells. DCFH-DA was dissolved in dimethyl sulfoxide (DMSO) and stored at -20°C in a 10 mM stock solution. MCF-7 cells and MDA-MB-231 cells were cultured with curcumin for 48 h, incubated with 10 *μ*M DCFH-DA for 30 minutes, washed 3 times with PBS, and immediately observed and imaged using a fluorescence microscope.

### 2.11. Measurement of MDA, Total Iron, and GSH Content

The cellular MDA, total iron, and GSH contents were determined using commercial kits. The MDA level is expressed as nmol/mg protein in relation to the cellular protein concentration. The GSH level is expressed as *μ*mol/gprot protein in relation to the cellular protein concentration.

### 2.12. Cell Morphology Observation

FITC fluorescent substance-labeled phalloidin specifically binds to F-actin in eukaryotic cells, thereby showing the distribution of the microfilament skeleton in the cell. Three days before being stained, cells were cultured on sterile glass coverslips. The coverslips were fixed with a 4% formaldehyde solution for 10 minutes, permeabilized with a 0.5% Triton X-100 solution for 5 minutes, and then incubated with FITC-labeled phalloidin in the dark for 30 minutes at room temperature. Cells were washed 3 times with PBS, and then, nuclei were counterstained for 30 seconds using a 100 nM DAPI solution, washed with PBS, inverted on a glass slide containing a drop of a Fluoromount-GTM water-soluble sealing solution, and permanently sealed with nail polish. Fluorescence was observed under a fluorescence microscope.

### 2.13. Detection of Lipid Peroxides in Cells

The Spy-LHP analogue Liperfluo was used for lipid peroxide detection. In organic solvents, such as ethanol, the specific oxidation of lipid peroxides causes intense fluorescence emission. The oxidized form shows little fluorescence in aqueous solution but produces strong fluorescence in fat-soluble fractions, such as cell membranes. Therefore, Liperfluo can be used for fluorescence imaging of lipid peroxides in living cells. First, 60 *μ*L DMSO was added to a test tube containing 50 *μ*g Liperfluo and uniformly mixed to prepare a Liperfluo solution at a concentration of 1 mmol/L. Then, 2 *μ*L of the Liperfluo solution was added to each well of a six-well plate and incubated for 30 minutes at 37°C in the dark. Cells were examined using a fluorescence microscope.

### 2.14. RNA Isolation and Quantitative Real-Time PCR

MCF-7 cells were randomly divided into two groups: the control and 40 *μ*M curcumin-treated groups. MDA-MB-231 cells were randomly divided into two groups: the control and 50 *μ*M curcumin-treated groups. Tested according to the methods of this laboratory, total RNA was isolated using TriPure isolation reagent (Roche, Mannheim, Germany), and DNA was removed from samples using DNase I (TaKaRa, Shiga, Japan). The purity of the isolated RNA was determined using a Nanodrop-2000 system (Thermo). RNA samples were used to prepare cDNA using a reverse transcriptase kit. Ten microliters of cDNA was analyzed using the SYBR Green PCR kit and a real-time fluorescence quantifier. The list of primers used for sequence validation is presented in the Supplement File (for the details of the primers used for sequence verification, please refer to Table 1 in the supplementary document). The primer pairs and probes were designed using the Primer3 software according to the recommendations described therein, and their specificity was determined with the Primer-BLAST program. Three analyses were performed for each sample. PCR was performed under the following conditions: 95°C for 10 minutes, 35 cycles at 95°C for 15 s, 60°C for 20 s, and 72°C for 30 s. Melting curve analysis confirmed the specificity of the amplification. The fold change of the target gene expression was normalized by the 2-fold abundance of *β*-actin mRNA^-*ΔΔ*Ct^ method.

### 2.15. Western Blot Analysis

Cells were extracted as lysates, and 20 *μ*g protein from each sample was subjected to 12% sodium dodecyl sulfate polyacrylamide gel electrophoresis (SDS-PAGE). The separated proteins were transferred to a nitrocellulose membrane (NC) filters (Pall, BioTrace NT, USA) and blocked with 5% skim milk in TBST (10 mmol/L Tris-HCl (pH 8.0), 150 mmol/L NaCl, and 0.1% Tween 20) at RT for 2 hours. The membranes were washed one time with TBST buffer and incubated with a suitable primary rabbit antibody (1 : 1000) specific for HO-1, GPX4, or Nrf2 at 4°C overnight. After washing four times with TBST, the immunoblotted membranes were incubated with a horseradish peroxidase-labeled goat antirabbit IgG-conjugated secondary antibody for 2 hours at room temperature. Finally, using a Pierce ECL substrate (Thermo Scientific, Waltham, Massachusetts, USA), protein bands were imaged on a chemiluminescence imaging analyzer (Tanon 5200, Shanghai, China).

### 2.16. Statistical Analysis

All experiments were performed independently at least three times, and the data are expressed as the mean ± standard error of the mean (SEM). The GraphPad PRISM software (Windows 5.02; GraphPad Software, Inc.) was used to test the significance of the data by the independent sample *t*-test, the statistical method for the treatment group using ZnPP used one-way analysis of variance, and *P* < 0.05 was deemed a statistically significant difference.

## 3. Results

### 3.1. Breast Cancer Cell Viability Is Inhibited by Curcumin Treatment

**Figure 1:**
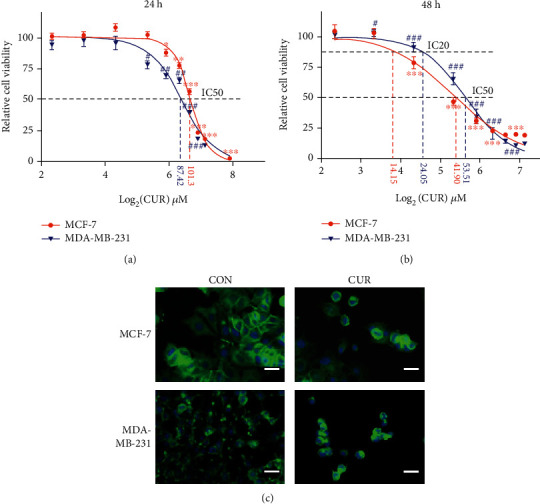
Breast cancer cell viability is inhibited by curcumin treatment. (a) Curcumin effectively inhibits the cell viability of cancer cells. Cancer cells were treated with the indicated concentrations of curcumin for 24 h. Cell viability was measured by the CCK-8 assay. Each point represents the mean value of three independent determinations; the error bars represent the SEM. ^∗^*P* < 0.05; ^∗∗^*P* < 0.01; ^∗∗∗^*P* < 0.001, compared to controls (MCF-7 cells). ^#^*P* < 0.05; ^##^*P* < 0.01; ^###^*P* < 0.001, compared to controls (MDA-MB-231 cells). (b) Curcumin effectively inhibits the cell viability of cancer cells. Cancer cells were treated with the indicated concentrations of curcumin for 48 h. Cell viability was measured by the CCK-8 assay. Each point represents the mean value of three independent determinations; the error bars represent the SEM. ^∗^*P* < 0.05; ^∗∗^*P* < 0.01; ^∗∗∗^*P* < 0.001, compared to controls (MCF-7 cells). ^#^*P* < 0.05; ^##^*P* < 0.01; ^###^*P* < 0.001, compared to controls (MDA-MB-231 cells). (c) MCF-7 cells were treated with 40 *μ*M curcumin for 48 h (CUR), and MCF-MB-231 cells were treated with 50 *μ*M curcumin for 48 h (CUR), with the control groups (CON) receiving the same volume of DMSO (curcumin solvent) as the treatment groups, and cell morphology was imaged using a fluorescence microscope (scale bar represents 100 *μ*m). Representative images from three independent experiments are shown. All the CON groups treated above received the same volume of reagent solvent as the treatment groups.

We first examined the effect of curcumin on breast cancer cell proliferation. Cell viability was used as a measure of cell proliferation. We treated breast cancer cells with curcumin at the same concentration gradient for 24 h and 48 h. After 24 hours of treatment, the IC50 values of curcumin in MCF-7 and MDA-MB-231 cells were approximately 101.3 *μ*M and 87.42 *μ*M, respectively (Figure 1(a)). After 48 h of treatment, the IC50 values of curcumin in MCF-7 and MDA-MB-231 cells were approximately 41.90 *μ*M and 53.51 *μ*M, respectively (Figure 1(b)). The research results of Ali et al. show that the IC50 value of curcumin at 24 h in human normal breast epithelial MCF-10A cells is approximately 190 *μ*M, and the IC50 value at 48 h is approximately 114 *μ*M [24]. Therefore, we selected the IC50 value of curcumin after 48 hours of breast cancer cell treatment for subsequent experiments. This choice allowed us to obtain sufficient biological information with less toxicity to and fewer side effects in normal breast epithelial cells, thereby laying a good foundation for studying the clinical anticancer effects of curcumin. Phalloidin staining was used to observe changes in cell morphology after 48 h of curcumin treatment. As shown in Figure 1(c), curcumin treatment caused significant changes in the morphology of breast cancer cells. The cell shape was significantly rounded, and the volume was greatly reduced.

### 3.2. Transcriptomic Analysis of the Effects of Curcumin on Breast Cancer Cells

**Figure 2:**
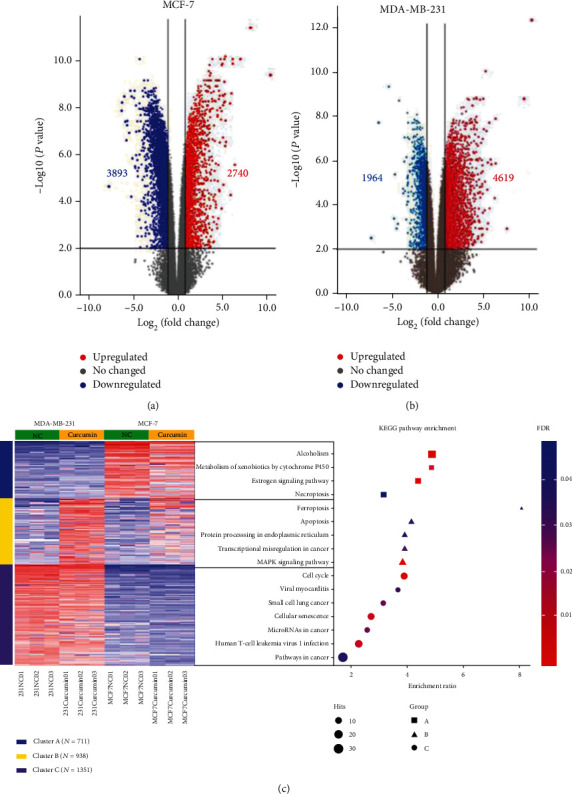
Differential expression and heat map hierarchical clustering analysis. (a, b) Volcano plots of the transcription levels of genes in control and 40 *μ*M curcumin-treated MCF-7 cells. Volcano plots of the transcription levels of genes in control and 50 *μ*M curcumin-treated MDA-MB-231 cells. (c) Differentially upregulated genes are defined by their fold change (log2fold change > 1) and FDR (−log10FDR > 1). (c) K-Means heat map hierarchical clustering and KEGG pathway enrichment analysis of the top 3000 DEGs among the 4 groups. The sequence of the enriched pathways represents the degree of enrichment. The area of each spot represents the number of genes contained in the pathway.

To systematically investigate the biological processes underlying the inhibition of breast cancer cell proliferation by curcumin, we sequenced the above curcumin-treated breast cancer cells by transcriptome sequencing. The results of the differential expression analysis revealed that a total of 2740 genes were upregulated, and 3893 genes were downregulated in the MCF-7 cell line, while in the MDA-MB-231 cell line, 4619 genes were upregulated, and 1964 genes were downregulated (Figures 2(a) and 2(b)). To further explore the key pathways shared by the two cell lines, we performed K-means clustering analysis. The results suggested that 3000 differentially expressed genes in the four groups formed three clusters. Cluster A is a specific upregulated gene groups (NC + curcumin) in the MCF-7 cell line, and Cluster C is a specific upregulated gene groups (NC + curcumin) in the MDA-MB-231 cell line. Cluster B is responsible for the genome of curcumin treatment effect (MDA-MB-231-curcumin+MCF7-curcumin). Therefore, we only focused on the signal pathways enriched in Cluster B; among them, the pathway with the highest enrichment ratio is ferroptosis, as shown in Figure 2(c).

### 3.3. Ferroptosis Network Analysis of Breast Cancer Cells Treated with Curcumin

**Figure 3:**
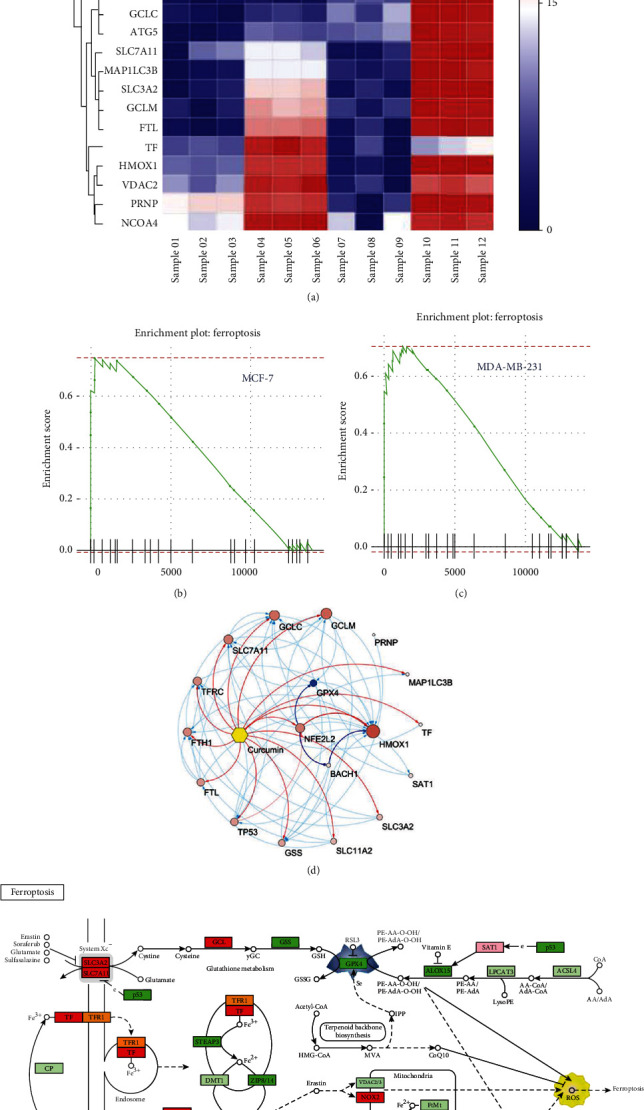
Ferroptosis network analysis of breast cancer cells treated with curcumin. (a) GSEA heat map analysis showing the ferroptotic pathway. Interactive heat map (GSEA) analysis was provided by NetworkAnalyst (https://www.networkanalyst.ca/NetworkAnalyst/home.xhtml). The value of 0-30 on the color scale bar of the heat map does not represent the relative fold change degree; each value is ranked on DE method used; the minimum value is 0, and the maximum value is 30, which has been explained by the literature provided by the official website [25]. (b, c) GSEA results based on KEGG pathways with enriched genes, indicating that the ferroptotic signaling pathway was upregulated by curcumin in both cell lines. (d) Cytoscape was used to construct a target gene interaction network diagram showing the ferroptotic pathways affected by curcumin according to the STRING database. (e) The ferroptotic pathway model was obtained from the KEGG pathway analysis results. The green boxes indicate genes that were downregulated by curcumin treatment. The red boxes indicate genes that were upregulated by curcumin treatment.

We generated a GSEA heat map based on analysis of the ferroptotic pathway form the results of the pathway enrichment analysis determined through NetworkAnalyst. As shown in Figure 3(a), we found that after treatment with curcumin at a specified concentration, MCF-7 cells exhibited greater gene activation than MDA-MB-231 cells, and the GSEA plots showed that the ferroptotic pathways in both cell lines were upregulated. Next, 14 curcumin targets were identified in the ferroptotic pathway according to the comparative toxicogenomics database(CTD), and in MCF-7 and MDA-MB-231 cells, these targets presented the same expression trends (Figures 3(b) and 3(c)). We used Cytoscape to construct a target gene interaction network diagram of curcumin in these ferroptotic pathways. Using the STRING database, we found that curcumin not only directly activates HO-1 but also upregulates its expression by activating nuclear factor-E2-related factor 2 (Nrf2). In addition, Nrf2 inhibits the expression of GPX4 (Figure 3(d)).The KEGG pathway analysis results indicated that the upregulation of HO-1 and downregulation of GPX4 directly trigger ROS production and that ferroptosis depends on the cytotoxicity induced by ROS (Figure 3(e)).

### 3.4. Curcumin Causes Iron Accumulation in Breast Cancer Cells to Induce Ferroptosis

**Figure 4:**
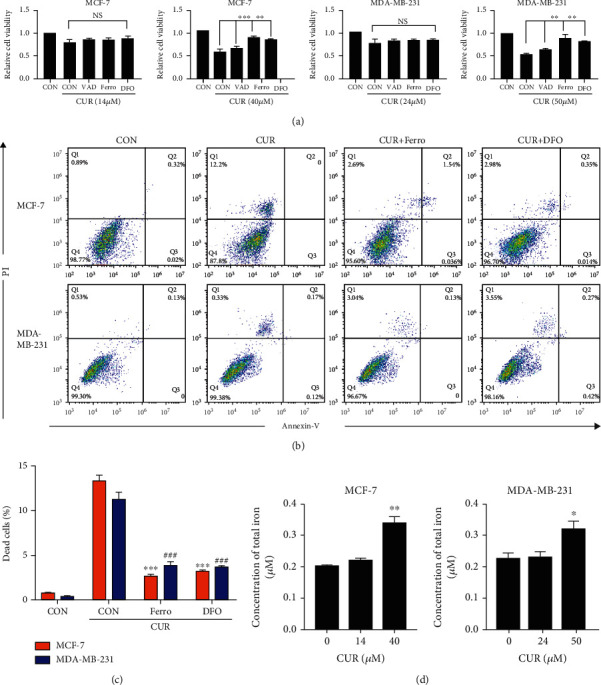
Curcumin causes iron accumulation in breast cancer cells to induce ferroptosis. (a) Cancer cells were preincubated with various inhibitors, z-VAD-fmk (VAD, 50 *μ*M), ferrostatin-1 (Ferro, 1 *μ*M), or deferoxamine (DFO, 50 *μ*M), for 2 h followed by curcumin (IC50) treatment for 48 h. Cell viability was assessed by the CCK-8 assay. All values are the mean ± SEM of three independent experiments. ^∗∗^*P* < 0.01; ^∗∗∗^*P* < 0.001, compared to CUR (curcumin) alone. NS: no significant difference compared to CUR (curcumin) alone. (b) Cancer cells were preincubated with various inhibitors, ferrostatin-1 (Ferro, 1 *μ*M), or deferoxamine (DFO, 50 *μ*M), for 2 h followed by curcumin (MCF-7: 40 *μ*M, MDA-MB-231: 50 *μ*M) treatment for 48 h. Cell death was assessed by Annexin V/PI staining. For Annexin V/PI staining, cells were collected, and the proportion of PI positive cells (dead) was determined by flow cytometry. Q1: nonapoptotic or ferroptotic cells (Annexin V negative/PI positive); Q2: late apoptotic and early necrotic cells (Annexin V positive/PI positive); Q3: early apoptotic cells (Annexin V positive/PI negative); Q4: viable cells (Annexin V negative/PI negative). (c) Use the FlowJo 10.5 software to perform statistical analysis on the experimental results of (b). All values are the mean ± SEM of three independent experiments. ^∗∗∗^*P* < 0.001, compared to CUR (curcumin) alone (MCF-7 cells). ^###^*P* < 0.001, compared to CUR (curcumin) alone (MDA-MB-231 cells). (d) After treating both cell lines with the IC20 (MCF-7: 14 *μ*m, MDA-MB-231: 24 *μ*m) and IC50 concentrations of curcumin for 48 h, the changes in the intracellular iron levels were measured according to the instructions of the kit. All the values are the mean ± SEM of three independent experiments. ^∗^*P* < 0.05; ^∗∗^*P* < 0.01, compared to CON (control) alone. All the CON (control) groups treated above received the same volume of reagent solvent as the treatment groups.

To confirm the validity of ferroptosis as the main cause of cell death, various pharmacological inhibitors of apoptosis (z-VAD-fmk) and ferroptosis (ferrostatin-1 and deferoxamine) were used to define the type of cell death induced by curcumin. As shown in Figure 4(a), when cells were treated with curcumin at the IC50 concentration, cell death induced by curcumin was significantly reduced in the presence of ferrostatin-1 and deferoxamine, but z-VAD-fmk had no such effect. At the same time, when the IC20 concentration of curcumin was used to treat cells, none of the pharmacological inhibitors had a significant effect on cell death induced by curcumin. Cell death was also determined by Annexin V/PI and analyzed by flow cytometry [23]. As shown in Figures 4(b) and 4(c), curcumin significantly increased the PI positive cell population but had little effect on Annexin V staining. The addition of ferrostatin-1and deferoxamine effectively suppressed curcumin-induced cell death. Next, we used the intracellular iron detection kit to further assess the changes in the total iron concentration in breast cancer cells after curcumin treatment. As shown in Figure 4(d), after 48 h of treatment with curcumin at the IC50 concentration, the total iron content of the cells was significantly increased, but there was no significant change at the IC20 concentration. These results indicate that the primary form of cell death caused by treatment of cells with the IC50 concentration of curcumin for 48 h can be attributed to ferroptosis.

### 3.5. RT-qPCR and Western Blotting Results Validate the RNA and Redox Proteins Related to the Ferroptotic Pathway

**Figure 5:**
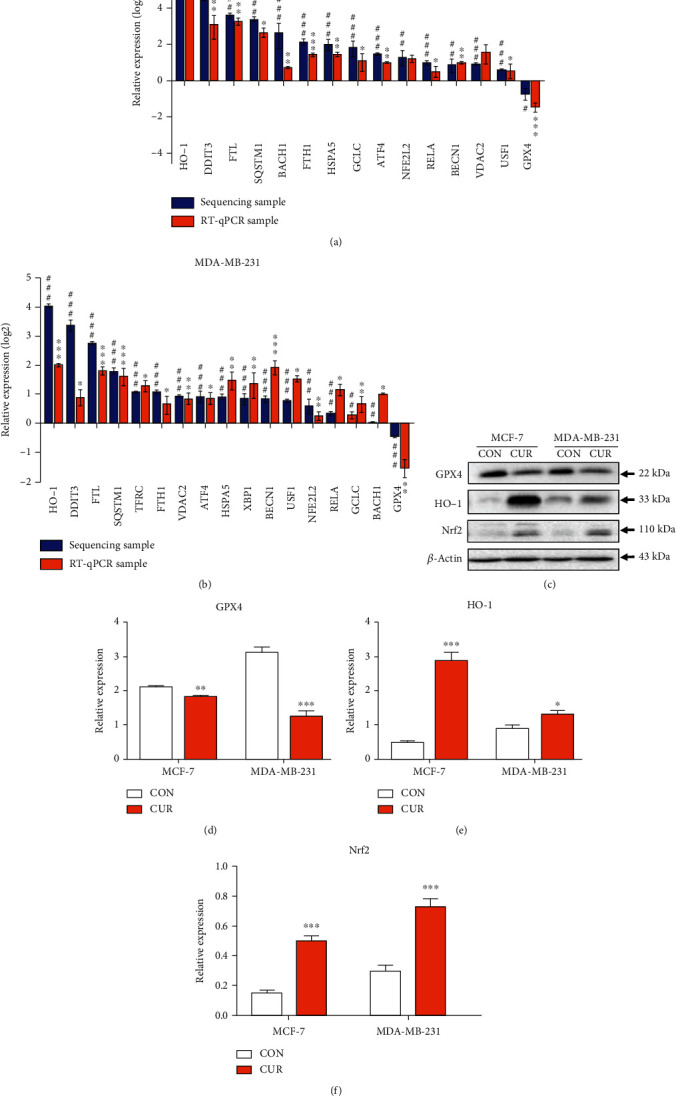
RT-qPCR verification of RNA associated with the ferroptotic pathway. (a) Total RNA extracted from MCF-7 cells treated with 40 *μ*M curcumin (CUR) for 48 hours was analyzed by RT-qPCR for gene expression analysis. The expression levels of mRNA were normalized to the level of *β*-actin. Each reported value represents the mean ± SEM from three independent experiments. ^∗^*P* < 0.05; ^∗∗^*P* < 0.01; ^∗∗∗^*P* < 0.001, compared to CON (control, RT-qPCR samples). ^#^*P* < 0.05; ^##^*P* < 0.01; ^###^*P* < 0.001, compared to CON (control, sequencing samples). (b) Total RNA extracted from MDA-MB-231 cells treated with 50 *μ*M curcumin (CUR) for 48 hours was analyzed by RT-qPCR for gene expression analysis. The expression levels of mRNA were normalized to the level of *β*-actin. Each reported value represents the mean ± SEM of three independent experiments. ^∗^*P* < 0.05; ^∗∗^*P* < 0.01; ^∗∗∗^*P* < 0.001, compared to CON (control, RT-qPCR samples). ^#^*P* < 0.05; ^##^*P* < 0.01; ^###^*P* < 0.001, compared to CON (control, sequencing samples). (c) MCF-7 cells were treated with vehicle or 40 *μ*M curcumin for 48 h, and MCF-MB-231 cells were treated with vehicle or 50 *μ*M curcumin for 48 h. Cells were then harvested for the western blot analysis. The representative figure shows one of the three independent experiments. (d) Quantitative results for the GPX4 expression in breast cancer cells as determined from greyscale analysis of (c) using the ImageJ software. The results are expressed as the means ± SEM of three independent experiments; ^∗∗^*P* < 0.01; ^∗∗∗^*P* < 0.001, compared to CON (control). (e) Quantitative results of the HO-1 expression in breast cancer cells determined by greyscale analysis of (c) using the ImageJ software. The results are expressed as the means ± SEM of three independent experiments; ^∗^*P* < 0.05; ^∗∗∗^*P* < 0.001, compared to CON (control). (f) Quantitative results of the Nrf2 expression in breast cancer cells determined by greyscale analysis of (c) using the ImageJ software. The results are expressed as the means ± SEM of three independent experiments; ^∗∗∗^*P* < 0.001, compared to CON (control). All CON (control) groups treated above received the same volume of reagent solvent as the treatment groups.

We next examined the gene expression associated with cellular redox reactions, intracellular iron homeostasis, autophagy, and endoplasmic reticulum stress, as shown in Figures 5(a) and 5(b), which have been shown to respond to ferroptotic agents. Curcumin upregulates genes involved in oxidative stress and ER stress, including HO-1, heat shock 70 kDa protein 5 (HSPA5), activating transcription factor 4 (ATF4), and DNA damage inducible transcript 3 (DDIT3). Curcumin also induced transcription factors, including BTB domain and CNC homolog 1 (BACH1), v-rel reticuloendotheliosis viral oncogene homolog A (RELA), upstream transcription factor 1 (USF1), NFE2-related factor 2 (NFE2L2), and the autophagy-related gene beclin 1 (BECN1). Cellular redox regulation and autophagy pathways were also responsive to curcumin, including glutamate-cysteine ligase catalytic subunit (GCLC), sequestosome 1 (SQSTM1), and X-box binding protein 1 (XBP1). At the same time, curcumin treatment caused a decrease in the GPX4 gene expression in both cells. The intracellular iron content is affected by the transferrin receptor and iron transporter. Studies have shown that the amount of intracellular transferrin is positively correlated with the degree of ferroptosis [26]. Curcumin upregulated FTL (encoding ferritin light chain), FTH1 (encoding ferritin heavy chain), and TFRC (encoding transferrin receptor) in MDA-MB-231 cells (Figure 5(b)). In MCF-7 cells, FTH1 and FTL were upregulated (Figure 5(a)).

Next, we used western blotting to determine the expression levels of GPX4, HO-1, and Nrf2 in both cell lines in response to curcumin treatment for 48 hours. The results showed that GPX4 was significantly downregulated compared its level in the control group (Figures 5(c) and 5(d)). HO-1 and Nrf2 were significantly upregulated compared with their levels in the control group (Figures 5(c)–5(f)), and Figures 5(c)–5(e) show that HO-1 in MCF-7 cells was upregulated to a greater extent than in MDA-MB-231 cells.

### 3.6. Curcumin Induces ROS Production in the Breast Cancer Cells

**Figure 6:**
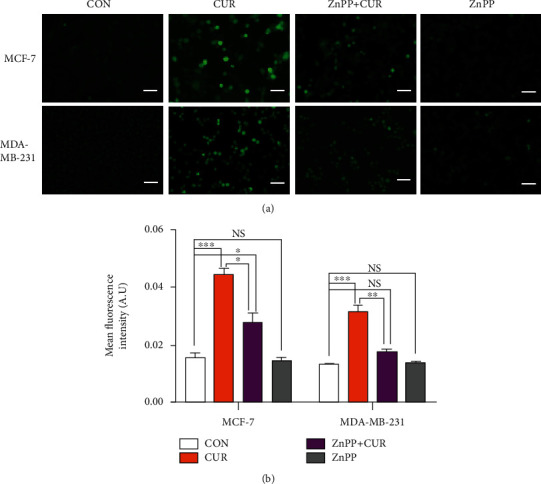
Determination of the ROS levels by fluorescence intensity measurements using a fluorescence microscope. (a) MCF-7 cells were treated with vehicle (CON, DMSO), curcumin (CUR, 40 *μ*M), or a mixture of 40 *μ*M curcumin and zinc protoporphyrin 9 (ZnPP, 10 *μ*M) for 48 hours. MDA-MB-231 cells were treated with vehicle (CON, DMSO), curcumin (CUR, 50 *μ*M), or a mixture of 50 *μ*M curcumin and zinc protoporphyrin 9 (ZnPP, 10 *μ*M) for 48 hours. After incubation with 5 *μ*M DCFH-DA, cells were washed and examined by a fluorescence microscopy (scale bar represents 200 *μ*m). Representative images from three independent experiments are shown. (b) The results of ROS upregulation in breast cancer cells were determined by fluorescence analysis using the ImageJ software. The results are expressed as the means ± SEM of three independent experiments;^∗^*P* < 0.05; ^∗∗^*P* < 0.01; ^∗∗∗^*P* < 0.001. NS: no significant difference compared to CON alone. All CON groups treated above received the same volume of reagent solvent as the treatment groups.

Ferroptosis cytotoxicity depends on ROS. To investigate whether upregulation of the HO-1 expression and downregulation of the GPX4expression cause inevitable ROS generation in the ferroptotic pathway, using DCFH-DA staining, we examined the intracellular ROS content after 48 hours of curcumin treatment. As shown in Figures 6(a) and 6(b), the ROS content in MCF-7 and MDA-MB-231 cells was significantly increased in response to curcumin treatment compared to the control group. At the same time, ZnPP treatment significantly prevented the increase in ROS levels caused by curcumin.

### 3.7. Marked Upregulation of HO-1 Is the Primary Factor for Curcumin-Induced Ferroptosis

**Figure 7:**
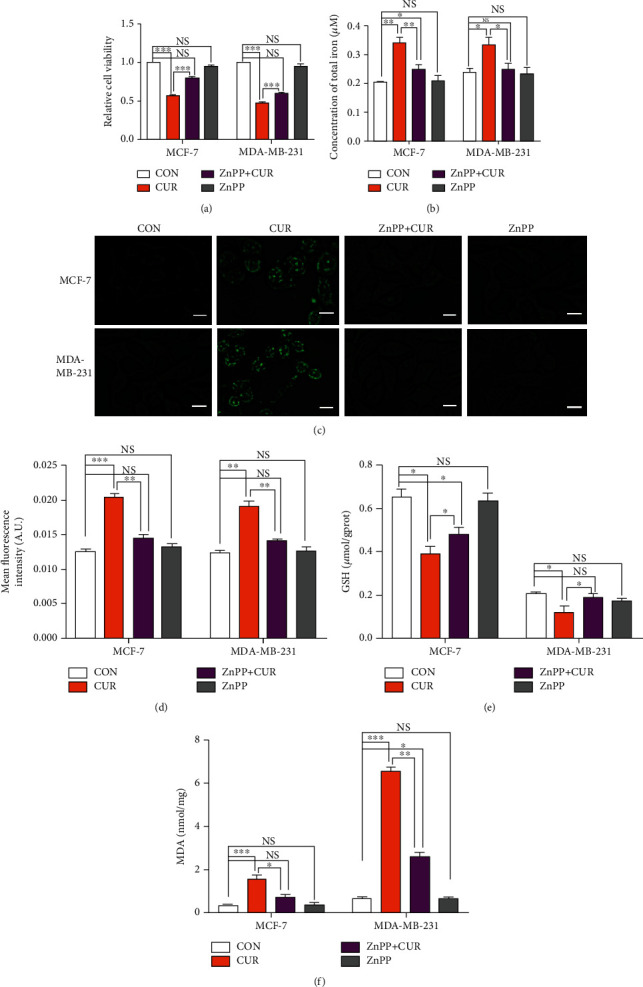
Curcumin upregulates the expression of HO-1 in breast cancer cells and triggers ferroptosis, as verified by ZnPP treatment. (a) Cell viability after treatment and under basal conditions (i.e., untreated cells) as measured by the CCK-8 assay. MCF-7 cells were treated with vehicle (CON, DMSO), curcumin (CUR, 40 *μ*M), or a mixture of 40 *μ*M curcumin and zinc protoporphyrin 9 (ZnPP, 10 *μ*M) for 48 hours. MDA-MB-231 cells were treated with vehicle (CON, DMSO), curcumin (CUR, 50 *μ*M), or a mixture of 50 *μ*M curcumin and zinc protoporphyrin 9 (ZnPP, 10 *μ*M) for 48 hours. The results are expressed as the means ± SEM of three independent experiments. ^∗∗∗^*P* < 0.001. NS: no significant difference compared to CON alone. (b) Iron assay kit to detect changes in the total iron content in cells. The cell treatment method is the same as for (a). The results are expressed as the means ± SEM of three independent experiments. ^∗^*P* < 0.05 and ^∗∗^*P* < 0.01. NS: no significant difference compared to CON alone. (c) Using Liperfluo to detect changes in the lipid peroxide content in cells. The cell treatment method was the same as for (a). After incubation with 2 *μ*M Liperfluo, cells were washed and examined by a fluorescence microscopy (scale bar represents 50 *μ*m). Representative images from three independent experiments are shown. (d) The results of lipid peroxide upregulation in breast cancer cells were determined by fluorescence analysis using the ImageJ software. The results are expressed as the means ± SEM of three independent experiments; ^∗∗^*P* < 0.01; ^∗∗∗^*P* < 0.001. NS: no significant difference compared to CON alone. All the CON groups treated above received the same volume of reagent solvent as the treatment group. (e) The GSH detection kit was used to detect changes in the GSH content in cells. The cell treatment method is the same as for (a). The results are expressed as the means ± SEM of three independent experiments. ^∗^*P* < 0.05; NS: no significant difference compared to CON alone. (f) The MDA detection kit was used to detect changes in the MDA content in cells. The cell treatment method is the same as for (a). The results are expressed as the means ± SEM of three independent experiments. ^∗^*P* < 0.05; ^∗∗^*P* < 0.01; ^∗∗∗^*P* < 0.001. NS: no significant difference compared to CON alone. All CON groups treated above received the same volume of reagent solvent as the treatment group.

To determine whether HO-1 is a key gene for curcumin-induced ferroptosis, we used a specific inhibitor of HO-1. The use of ZnPP to verify the role of HO-1 in cell ferroptosis has proven very effective [27]. Treatment with ZnPP effectively alleviated curcumin-induced cancer cell death, as shown in Figure 7(a). ZnPP also significantly reduced the intracellular iron accumulation induced by curcumin (Figure 7(b)). In addition, for live cell imaging, we used Liperfluo (L248), which reduces lipid hydrogen peroxide to its hydroxyl homologues, producing fluorescent products. Liperfluo fluorescence reliably reflects intracellular sites of lipid hydroperoxide accumulation [28]. We found that breast cancer cells accumulated lipid-reactive lipid hydrogen peroxide for 48 hours after curcumin treatment. The accumulation of lipid hydroperoxide, as shown in these cells, is considered the primary feature of ferroptosis. Subsequently, accumulation of lipid hydroperoxide was partially inhibited by ZnPP (Figures 7(c) and 7(d)). GSH is an important reducing agent involved in cellular redox reactions and participates in the ferroptotic pathway. Treatment with curcumin caused significant inhibition of the intracellular GSH levels, but treatment with ZnPP reversed this phenomenon (Figure 7(e)). Free radicals induce lipid peroxidation, and the final oxidation product is malonaldehyde, which is cytotoxic. After curcumin treatment of breast cancer cells for 48 hours, the intracellular MDA content was significantly increased, and compared to the curcumin treatment group, ZnPP treatment significantly reduced the MDA content (Figure 7(f)). Taken together, these results indicate that in response to ZnPP inhibition of HO-1, curcumin-induced breast cancer cell death and cell lipid peroxidation are significantly attenuated, and the intracellular glutathione levels are increased. Therefore, HO-1 plays a significant role in promoting curcumin-induced breast cancer cell ferroptosis.

## 4. Discussion

In general, the consumption of curcumin is considered safe and healthy. As per JECFA (The Joint FAO/WHO Expert Committee on Food Additives) and EFSA (European Food Safety Authority) reports, the adequate daily intake value of curcumin is from 0-3 mg·kg^−1^ [29, 30]. According to the results of Ramachandran et al., MCF-7 cells are more sensitive to curcumin than normal human breast epithelial cells (MCF-10A). After curcumin treatment, MCF-10A cells break down curcumin and retain relatively little drug in the medium, thereby reducing the cytotoxic effect of MCF-10A cells. Curcumin induced a significantly higher percentage of Annexin V positive apoptotic cells in MCF-7 than in MCF-10A cells. In addition, among the apoptosis-related genes identified by microarray hybridization, curcumin treatment of MCF-7 cells upregulated apoptosis regulatory factors, including Bcl-w, caspase-2 precursor, caspase-3, and caspase-4. However, curcumin-induced gene expression changes were significantly reduced in MCF-10A cells, and the changes were always less than 2-fold [31]. These results indicate that curcumin induces breast cancer cell apoptosis while having relatively few side effects on human normal breast epithelial cells.

In this study, RNA was sequenced and quantified from breast cancer cells with and without curcumin treatment. Bioinformatics analysis after RNA sequencing showed that both the apoptotic and ferroptotic pathways were enriched by KEGG pathway analysis (Figure 2(c)). However, it can be seen in the figure that the degree of enrichment of the ferroptotic pathway was greater than the apoptosis pathway. To further clarify the form of cell death caused by treatment of cells with the IC50 concentration of curcumin for 48 hours, apoptosis inhibitors and ferroptosis inhibitors were used (Figure 4). The results showed that compared to the curcumin treatment group, the addition of ferroptosis inhibitors significantly improved cell viability and inhibited cell death, while apoptosis inhibitors had no such effect. Based on the above data, it can be inferred that ferroptosis is the primary pathway of death in this experiment. We conducted a series of validation tests and found that cancer cells treated with curcumin for 48 hours showed increased ROS levels and significantly reduced GPX4, cell lipid peroxidation, and iron accumulation levels. Class I ferroptotic inducers (e.g., erastin and sulfasalazine) cause cellular GSH depletion and a redox status imbalance [32]. Class II ferroptotic inducers, especially GPX4 inhibitors (e.g., RSL3 and DPI derivatives), can cause fatal levels of lipid peroxidation [33]. The results of our experiments were consistent with the treatment results of these recognized ferroptotic compounds. Therefore, we can be certain that, in these experiments, curcumin-treated breast cancer cells underwent ferroptosis.

An increasing number of studies have shown that HO-1 plays a key role in ferroptosis and plays a pathogenic role in the development of many diseases [34, 35]. Patients with Alzheimer's disease exhibit increased lipid peroxidation levels, which may be related to increased HO-1 and iron accumulation [36]. Several HO-1 inhibitors have been developed and widely and effectively used. ZnPP is a heme analog composed of protoporphyrin IX and metal Zn [37]. Due to its similar chemical structure to heme, it is possible to competitively inhibit enzymatic activity by occupying the heme binding site of heme oxygenase. ZnPP has been widely used in studying HO-1 in ferroptosis. In breast cancer cells, ZnPP not only inhibits BAY-induced cell ferroptosis but also significantly prevents the increase of unstable iron pools caused by BAY and h-HO-1 overexpression [27]. The carcinogenic RAS selective lethal small molecule erastin induces ferroptosis. In HT-1080 fibrosarcoma cells, erastin induces the HO-1 expression in a time- and dose-dependent manner, and erastin-induced cellular ferroptosis is inhibited by ZnPP in a manner similar as ferrostatin-1, which has been identified as an inhibitor of ferroptosis [28].

HO-1 is a rate-limiting enzyme in the degradation of heme. Some studies have shown that the induction of HO-1 has antitumor activity. The induction of HO-1 in breast cancer suppressed proliferation and invasion through reducing intracellular ROS [38, 39]. Overexpression of HO-1 in hepatomas reduced cell migration and xenograft tumor growth [40]. In addition, induction of HO-1 by chemopreventive agents such as curcumin and sulforaphane inhibits tumorigenesis via increasing antioxidant response genes [41, 42]. Heme can be degraded into bilirubin, CO, and Fe^2+^. Upregulation of HO-1 enhances the degradation of heme and the synthesis of ferritin, altering the intracellular iron distribution [43]. Iron overload can promote Fenton reactions and ROS production. Excessive ROS generation leads to peroxidation and oxidative damage to neighboring lipids, DNA, and proteins, eventually leading to ferroptosis [44]. The metabolism of iron in cells is regulated by related ferritin. When the regulation capacity of ferritin is disturbed by strong oxidative stress, it causes uncontrolled release of large amounts of iron, eventually leading to excessive accumulation of iron in the cell and subsequent lipid peroxidation [45]. Activation of iron metabolism-related proteins promotes the development of ferroptosis [26]. In our study, the gene expression of the transferrin receptor and iron transporter in breast cancer cells was significantly upregulated, and cellular ROS and cellular lipid peroxidation were intensified. It can be concluded that overexpression of HO-1 causes intracellular iron metabolic disorders and cellular lipid peroxidation, leading to ferroptosis. Previous studies have detailed the dual role of HO-1 in cells, and its role in ferroptosis may be cell type and stimulus specific [46]. In a recent study of kidney damage related to rhabdomyolysis, curcumin was found to inhibit renal tubular cell ferroptosis by activating HO-1 [47]. This finding means that the exact regulatory mechanism of curcumin in ferroptosis needs to be further studied and that the role of HO-1 in ferroptosis still needs to be elaborated. Furthermore, the application of curcumin in cancer chemotherapy needs far more research support.

In addition, GPX4 is an essential regulator of ferroptotic cancer cell death that provides antioxidants to counteract lipid peroxidation and is the only enzyme that reduces lipid hydroperoxides in biofilms [48]. GPX4 is a key factor in maintaining cell redox balance [49]. Analysis of mass spectrometry-based proteomics data from an affinity pull-down experiment ranked GPX4 as the top protein target for (1S,3R)-RSL3 [50]. Friedmann et al. provided direct genetic evidence that knocking out GPX4 leads to cell death in a pathologically related form of ferroptosis [51]. Glyoxalases (Glo1 and Glo2) are involved in the glycolytic pathway by detoxifying the reactive methylglyoxal (MGO) into d-lactate in a two-step reaction using glutathione (GSH) as cofactor. Inhibitors of glyoxalases are considered anti-inflammatory and antitumor agents. Curcumin inhibits Glo1, resulting in nontolerable levels of MGO and GSH. As a result, various metabolic pathways are disturbed so that, for example, cellular ATP and GSH content are depleted [52]. The depletion of cellular ATP and GSH may in turn decrease cell survival. GPX4 reduces membrane hydroperoxide through GSH, and GPX4 and GSH appear together to become the main determinant of the balance between cell proliferation and death. The inactivation of GPx4 or the depletion of GSH in the cell can lead to a new type of cell death that depends on lipid peroxidation, which is called ferroptosis [53]. Therefore, GPX4 and GSH play an important regulatory role in ferroptosis.

In both the sequencing and RT-qPCR results in our study, curcumin treatment significantly downregulated GPX4 and upregulated HO-1. It is worth noting that 48 hours after curcumin treatment, the upregulation of the HO-1 expression in MCF-7 cells was dramatic. The degree of GPX4 downregulation was similar between the two cell lines, but the degree of ferroptosis in the two cells was different, indicating that GPX4 is not the most important regulator in the process of curcumin-induced breast cancer cell ferroptosis. Therefore, we believe that GPX4 is a basic effector of curcumin, but HO-1 seems to be more responsible for the difference in the inhibitory effects of curcumin observed in the two cell lines. HO-1 is the limiting factor for curcumin to exert its therapeutic role and thus is the preferred target gene for this study. Although the comparative toxicogenomics database suggests HO-1 as a direct target gene for curcumin, in our opinion, the activation of HO-1 also depends on additional transcription factors, including Nrf2. Nrf2 is a major regulator of cellular antioxidant and electrophilic stress defense responses, and HO-1 is one of its target genes [54]. Due to its binding to the cytoskeleton-related protein Keap1, the inactive form of Nrf2 partially or fully localizes in the cytoplasm. The activation of Nrf2 is considered an important molecular target for many chemopreventive and cytoprotective agents. Nrf2 protects cells against oxidative stress through ARE-directed induction of several phase 2 detoxifying and antioxidant enzymes, particularly HO-1. Some studies have reported that Nrf2 inhibits ferroptosis [55, 56], but the results from study by Cao et al. have shown that even when Nrf2 induces increased intracellular glutathione content, it only weakly protects cells from ferroptosis [57].

## 5. Conclusions

Based on the evidence that curcumin has a significant inhibitory effect on breast cancer cells, this study analyzed the possible genetic pathways of curcumin targeting breast cancer cells using RNA sequence technology. Ferroptosis, a novel pathogenic mechanism screened from high-throughput data, was verified in our in vitro results. The results of our current study suggest that curcumin triggers the molecular and cytological features of ferroptosis in breast cancer cells by upregulating HO-1 and downregulating GPX4. In subsequent experiments, it was obvious that ferroptosis occurred in response to curcumin treatment. After treatment with the HO-1 specific inhibitor ZnPP, compared to the curcumin-treated group, these phenomena were partially reversed. Therefore, these data highlight that curcumin triggers the molecular and cytological features of ferroptosis in breast cancer cells and that HO-1 potentiates curcumin-induced ferroptosis. Of note, this study only examined the anticancer effects of curcumin on two representative breast cancer cell lines. Whether curcumin can be used as a broad-spectrum antibreast cancer drug remains unknown, and more extensive follow-up investigations are needed. Our study only provides the basis of molecular data for the mechanism of action of curcumin.

## Data Availability

The datasets generated for this study can be found in NCBI. The BioProject accession of this study is PRJNA613560.

## Supplementary Materials

Supplementary Materials Table 1: the list of the primers used for sequence validation.
