# Supplementary material for: Transcriptome Investigation and In Vitro Verification of Curcumin-Induced HO-1 as a Feature of Ferroptosis in Breast Cancer Cells
Source: Oxid Med Cell Longev. 2020 Nov 19;2020:3469840. doi: 10.1155/2020/3469840 (PMC7691002; doi:10.1155/2020/3469840)
Supplement: Supplementary Materials — Table 1: the list of the primers used for sequence validation. [file 3469840.f1.docx]

**Supplementary Materials:**

Table1: The list of the primers used for sequence validation

| *Gene* | *GenBank Accession* | Primer sequence (5′-3′) |
| --- | --- | --- |
| *ATF4* | *NM_182810* | Forward ATGACCGAAATGAGCTTCCTG  Reverse GCTGGAGAACCCATGAGGT |
| *β-actin* | *NM_001101* | Forward CATGTACGTTGCTATCCAGGC  Reverse CTCCTTAATGTCACGCACGAT |
| *BACH1* | *NM_206866* | Forward CCGCTTCAGTCTCTACCATATC  Reverse ATGCCACTGTATTCTGAGTCC |
| *BECN1* | *NM_003766* | Forward GGTGTCTCTCGCAGATTCATC  Reverse TCAGTCTTCGGCTGAGGTTCT |
| *DDIT3* | *NM_001195055* | Forward GGAAACAGAGTGGTCATTCCC  Reverse CTGCTTGAGCCGTTCATTCTC |
| *FTL* | *NM_000146* | Forward CAGCCTGGTCAATTTGTACCT  Reverse GCCAATTCGCGGAAGAAGTG |
| *FTH1* | *NM_002032* | Forward CGAGGTGGCCGAATCTTCC  Reverse GTTTGTGCAGTTCCAGTAGTGA |
| *GCLC* | *NM_001197115* | Forward GGAGACCAGAGTATGGGAGTT  Reverse CCGGCGTTTTCGCATGTTG |
| *GPX4* | *NM_001039847* | Forward GAGGCAAGACCGAAGTAAACTAC  Reverse CCGAACTGGTTACACGGGAA |
| *HO-1* | *NM_002133* | Forward TTCAGCATCCTCAGTTCC  Reverse CCGTGTCAACAAGGATAC |
| *HSPA5* | *NM_005347* | Forward CATCACGCCGTCCTATGTCG  Reverse CGTCAAAGACCGTGTTCTCG |
| *NFE2L2* | *NM_001145412* | Forward TCCAGTCAGAAACCAGTGGAT  Reverse GAATGTCTGCGCCAAAAGCTG |
| *RELA* | *NM_001145138* | Forward ATGTGGAGATCATTGAGCAGC  Reverse CCTGGTCCTGTGTAGCCATT |
| *SQSTM1* | *NM_001142299* | Forward GACTACGACTTGTGTAGCGTC  Reverse AGTGTCCGTGTTTCACCTTCC |
| *TFRC* | *NM_003234* | Forward GGCTACTTGGGCTATTGTAAAGG  Reverse CAGTTTCTCCGACAACTTTCTCT |
| *USF1* | *NM_207005* | Forward TCCCAGACTGCTCTAT GGAGA  Reverse CGGTGGTTACTCTGCC GAAG |
| *VDAC2* | *NM_001184783* | Forward GCTACAGGACTGGGGACTTC  Reverse AATGCCAAAACGAGTGCAGTT |
| *XBP1* | *NM_001079539* | Forward CCCTCCAGAACATCTCCCCAT  Reverse ACATGACTGGGTCCAAGTTGT |
